# Causal effects of immune cell surface antigens and functional outcome after ischemic stroke: a Mendelian randomization study

**DOI:** 10.3389/fimmu.2024.1353034

**Published:** 2024-03-18

**Authors:** Weiming Sun, Jiawei Gui, Keqi Wan, Yize Cai, Xiangli Dong, Guohua Yu, Chafeng Zheng, Zhen Feng, Lang Shuai

**Affiliations:** ^1^ Department of Rehabilitation Medicine, The First Affiliated Hospital, Jiangxi Medical College, Nanchang University, Nanchang, China; ^2^ The First Clinical Medical College, Jiangxi Medical College, Nanchang University, Nanchang, China; ^3^ Postdoctoral Innovation Practice Base, The First Affiliated Hospital, Jiangxi Medical College, Nanchang University, Nanchang, China; ^4^ HuanKui Academy, Jiangxi Medical College, Nanchang University, Nanchang, China; ^5^ School of Public Policy, Nanchang University, Nanchang, China; ^6^ Department of Psychosomatic Medicine, The Second Affiliated Hospital, Jiangxi Medical College, Nanchang University, Nanchang, China

**Keywords:** immune cell, surface antigens, ischemic stroke, prognosis, Mendelian randomization

## Abstract

**Objective:**

While observational studies link immune cells with post-stroke functional outcome, the underlying immune mechanisms are not well understood. Immune cell surface antigens are actively involved in the biological behavior of immune cells, investigating immune cell surface antigens could deepen our comprehension of their role and biological processes in stroke recovery. Therefore, we aimed to investigate the immunological basis of stroke outcome by exploring the causal relationship between immune cell surface antigens and functional outcome after ischemic stroke in a Mendelian randomization study.

**Methods:**

Genetic variants related to immune cell surface antigens and post-stroke functional outcome were selected for two-sample Mendelian randomization (MR) analysis. 389 fluorescence intensities (MFIs) with surface antigens were included. Inverse variance weighted (IVW) modeling was used as the primary MR method to estimate the causal effect of exposure on the outcome, followed by several alternative methods and sensitivity analyses. Additional analysis of the association between immune cell surface antigens and risk of ischemic stroke for assessment of collider bias.

**Results:**

We found that suggestive associations between CD20 on switched memory B cell (OR = 1.16, 95% CI: 1.01-1.34, p** =** 0.036) and PDL-1 on monocyte (OR = 1.32, 95% CI: 1.04-1.66, p = 0.022) and poor post-stroke functional outcome, whereas CD25 on CD39+ resting Treg (OR = 0.77, 95% CI: 0.62-0.96, p = 0.017) was suggestively associated with good post-stroke functional outcome.

**Conclusion:**

The elevated CD20 on switched memory B cell, PDL-1 on monocyte, and CD25 on CD39+ resting Treg may be novel biomarkers and potential causal factors influencing post-stroke functional outcome.

## 1 Introduction

Ischemic stroke, accounting for 62.4% of stroke events in 2019, is the predominant stroke type with significant long-term neurological impairment and high mortality (1). The complex and poorly understood pathogenesis of ischemic stroke leads to uncertain treatment strategies. Despite available treatments like thrombus removal, their limited effectiveness and narrow therapeutic window often result in an unfavourable outcome for many patients (2). Hence, there is an urgent need to identify novel biomarkers and therapeutic targets for ischemic stroke treatment.

Recent studies indicate that ischemic stroke triggers neuroinflammation, characterized by lymphopenia and dysfunction of immune cells, highlighting the critical role of the immune response in stroke outcome (3, 4). Understanding how immunity influences neurological recovery is thus essential. The characteristics of immune cells in stroke patients mirror the body’s immune status and are strongly linked to prognosis (5, 6). For instance, regulatory T cells (Tregs), a crucial subset of immunosuppressive T cells, are believed to modulate immune responses in ischemic strokes, impacting prognosis (7, 8). CD4+ Treg levels at admission predict the modified Rankin Scale (mRS) score three months post-stroke, correlating positively with outcome (9). Immune cell surface antigens, key in immune cell differentiation, activation, and signaling, determine immune cell properties, indicating changes in function and status and reflecting their phenotype. Targeting specific surface antigens on immune cells could improve ischemic stroke therapy outcomes (10). Yet, the exact relationship between these antigens and the post-stroke functional outcome remains to be elucidated with existing studies potentially affected by reverse causation and confounding factors. Given that specific immune cell surface antigens might impact post-stroke functional outcome, further research is essential to deepen our understanding.

Mendelian randomization (MR), utilizing germline genetic variants to investigate the causal effects of exposures on outcome, is a pivotal methodology in epidemiological etiological inference (11–13). The general independence of genetic variations from environmental influences and outcomes provides us with a favorable tool to study the causality of several complex exposures and outcomes. Therefore, we performed a two-sample MR framework using genome-wide association studies (GWAS) data to explore the potential causal associations between immune cell surface antigens and post-stroke functional outcome.

## 2 Methods

### 2.1 Study design

**Figure 1:**
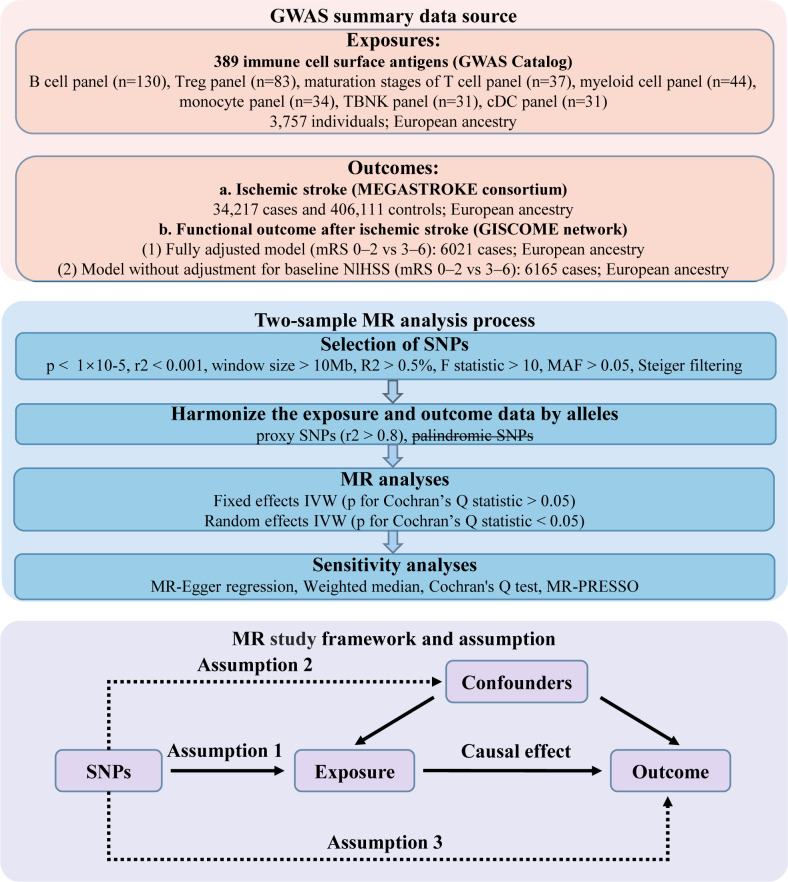
Design of the present Mendelian randomization study of the associations between immune cell surface antigens and post-stroke functional outcome.

In this study, we utilized GWAS summary statistics for a two-sample MR analysis to determine the causal effect of immune cell surface antigens on post-stroke functional outcome (**Figure 1**). Instrumental variables (IVs) in MR must meet three core assumptions: (1) association with the exposure; (2) independence from confounders; (3) influence on the outcome exclusively through the exposure. Our analysis relied on publicly available GWAS summary statistics from cohorts primarily of European ancestry. We carefully reviewed the original studies, and found that sample overlap was negligible. An overview of the GWAS summary data sources is presented in **Table 1**.

**Table 1:** An overview of the GWAS summary data sources in this study.

Traits	Data source	Sample size or cases/controls	Number of SNPs	Ancestry	Publication year	PMID
**389 immune cell surface antigens**	GWAS Catalog	3,757 individuals	∼22 million	European	2020	32929287
**Ischemic stroke**	MEGASTROKE consortium	34,217 cases; 406,111 controls	∼8.3 million	European	2018	29531354
**Functional outcome after ischemic stroke**	GISCOME network	6021 cases	∼8.5 million	European	2019	30796134

### 2.2 Genetic instruments for immune cell surface antigens

In this MR study, we sourced genetic variants linked to immune cell surface antigens (measured by median fluorescence intensities, MFIs) from the publicly available GWAS Catalog (https://www.ebi.ac.u/gwas/home). The initial genome-wide GWAS analysis utilized data from 3,757 individuals of European ancestry (14).

The MFI represents the median expression level of a fluorescent-conjugated antibody bound to a cell, directly proportional to the median quantity of antigen expressed in that cell. The distribution was normalized for overall and daily fluctuations to control batch effects in MFIs. A total of 389 MFIs with surface antigens were included in seven panels (maturation stages of T cell, Treg, TBNK, DC, B cell, monocyte, and myeloid cell, respectively). All immune cells used to measure MFIs were collected from the participant’s peripheral blood. Details of the 389 MFIs are listed in **Supplementary Table S1**. Genetic variants were screened based on the following conditions: (1) Single nucleotide polymorphisms (SNPs, refer to DNA sequence polymorphisms caused by variation in a single nucleotide at the genomic level) associated with MFIs of immune cell surface antigens (*P<* 1×10-5) and not in linkage disequilibrium (LD) with other SNPs (r^2^< 0.001 within a clumping window of 10000 kb); (2) a phenotypic variance explained (PVE, evaluated using the R^2^) > 0.5% and a F statistic >10; the F statistic was calculated as follows: $\frac{\text{R}2\left(\text{N}-\text{K}-1\right)}{\text{K}\left(1-\text{R}2\right)}$ (R^2^, phenotypic variance explained; N, effective sample size; K, the quantity of genetic variants); (3) a minor allele frequency (MAF) > 0.05; (4) exclusion of SNPs associated with the outcome (*P<* 1×10-5). The remaining SNPs were utilized as IVs. Subsequently, we harmonized the alleles and effects between the exposure and outcome. When the SNPs were not identified in the outcome data, the proxy SNPs (r^2^ > 0.8) from 1000 genomes European reference data were used to replace them. The SNPs that have palindromic alleles with intermediate allele frequencies (MAF > 0.42) were removed. Furthermore, we applied Steiger filtering to exclude SNPs that explained more of the variance in the outcome than the exposure. In the reverse MR analysis, the screening criteria for IVs were the same as above.

### 2.3 Outcome data sources

We derived GWAS summary statistics for post-ischemic stroke functional outcome from the Genetics of Ischemic Stroke Functional Outcome (GISCOME) network (15), comprising 6,021 patients across 12 studies from Europe, Australia, and the United States (16). Participants were European ancestry and aged 18 or above. Post-stroke functional outcome refers to a person’s level of physical, mental and cognitive ability after a stroke, which includes a range of factors such as mobility, strength, coordination, speech, language, memory (15, 17). The main focus of research has been on unfavorable functional outcomes for stroke patients, including cognitive impairment or dementia, dependency, disability, motor impairment, psychological impairment (depression or anxiety) and death (18, 19). The mRS approximately 3 months post-stroke was selected to assess functional outcome. mRS assesses dependency of stroke patients and ranges from 0 (no symptoms) to 5 (completely dependent and bed ridden), and death was included in scale (mRS score = 6), which is a commonly used scale for measuring the degree of disability of people who have suffered a stroke or other causes of neurological disability. We classified a ‘poor’ outcome as an mRS score > 3 (2,280 cases) and a ‘good’ outcome as a score< 2 (3,741 cases). In our analyses, the mRS was analyzed as 2 dichotomous variables (score of 0–2 vs 3–6), and the results were adjusted for age, sex, ancestry, and baseline stroke severity as evaluated by the NIH Stroke Scale (NIHSS).

### 2.4 Assessment of collider bias

To assess whether the causal association between MFIs of immune cell surface antigens and functional outcome after ischemic stroke is attributable to collider bias, we also performed an MR analysis between immune cell surface antigens and the risk of ischemic stroke. The summary statistics were obtained from the MEGASTROKE consortium, which included 406,111 controls and 34,217 patients with ischemic stroke (20). Participants were drawn from 17 studies and were restricted to Europeans only. SNPs that met the MEGASTROKE criteria (n_cases > 50% and oevar_im*p >* 0.5) were selected for the MR analysis.

### 2.5 Statistical analysis

The inverse-variance weighted (IVW) method was adopted as the main MR analysis. To account for multiple hypothesis testing, we applied Bonferroni correction with a significance threshold of *P<* 1.285 × 10^-4^ (0.05/389), indicating statistical significance. We also considered results with p-values of 1.285 × 10-4 to 0.05 nominally significant. Sensitivity analyses were performed using the weighted median, MR-Egger regression, and MR-Pleiotropy Residual Sum and Outlier (MR-PRESSO). The weighted median method yields consistent estimates when over 50% of the weights originate from valid instrumental variables (21). MR-Egger regression, both for the intercept and slope, assessed directional pleiotropy and provided robust estimates adjusted for its presence (22). Specifically, MR Egger regression tests for the presence of directional pleiotropy by examining the intercept term and provides an approximately unbiased estimate of the causal effect of exposure on outcome by incorporating the intercept into the regression model (22). MR-PRESSO was utilized to detect and account for potential horizontal pleiotropy and to identify and exclude any outliers with such effects (23). In cases where pleiotropy and heterogeneity were absent, a significant result (*P<* 0.05) obtained via the IVW method was considered positive, provided that the effect estimates from other methods were consistent with those of the IVW method. Cochran’s Q statistic assessed heterogeneity among instrumental variables. If heterogeneity was present (*P<* 0.05), a random-effects IVW model was applied. For comparison, we conducted an MR analysis using GISCOME GWAS data without adjusting for baseline NIHSS. All statistical analyses were carried out using the MR-PRESSO (version 1.0) (23) and TwoSampleMR (version 0.5.7) (24) packages in the R software environment.

## 3 Results

### 3.1 Thirteen immune cell surface antigens as potential causal mediators of functional outcome after stroke in the main MR analysis

The number of SNPs as IVs generated by 389 MFIs of immune cell surface antigens for MR analysis ranged from 7 to 30, and all IVs passed Steiger filtering. Notably, all IVs exhibited F statistics exceeding 10, with a minimum F statistic of 19.54, indicating the significant effectiveness of these IVs (**Supplementary Table S2**).

**Figure 2:**
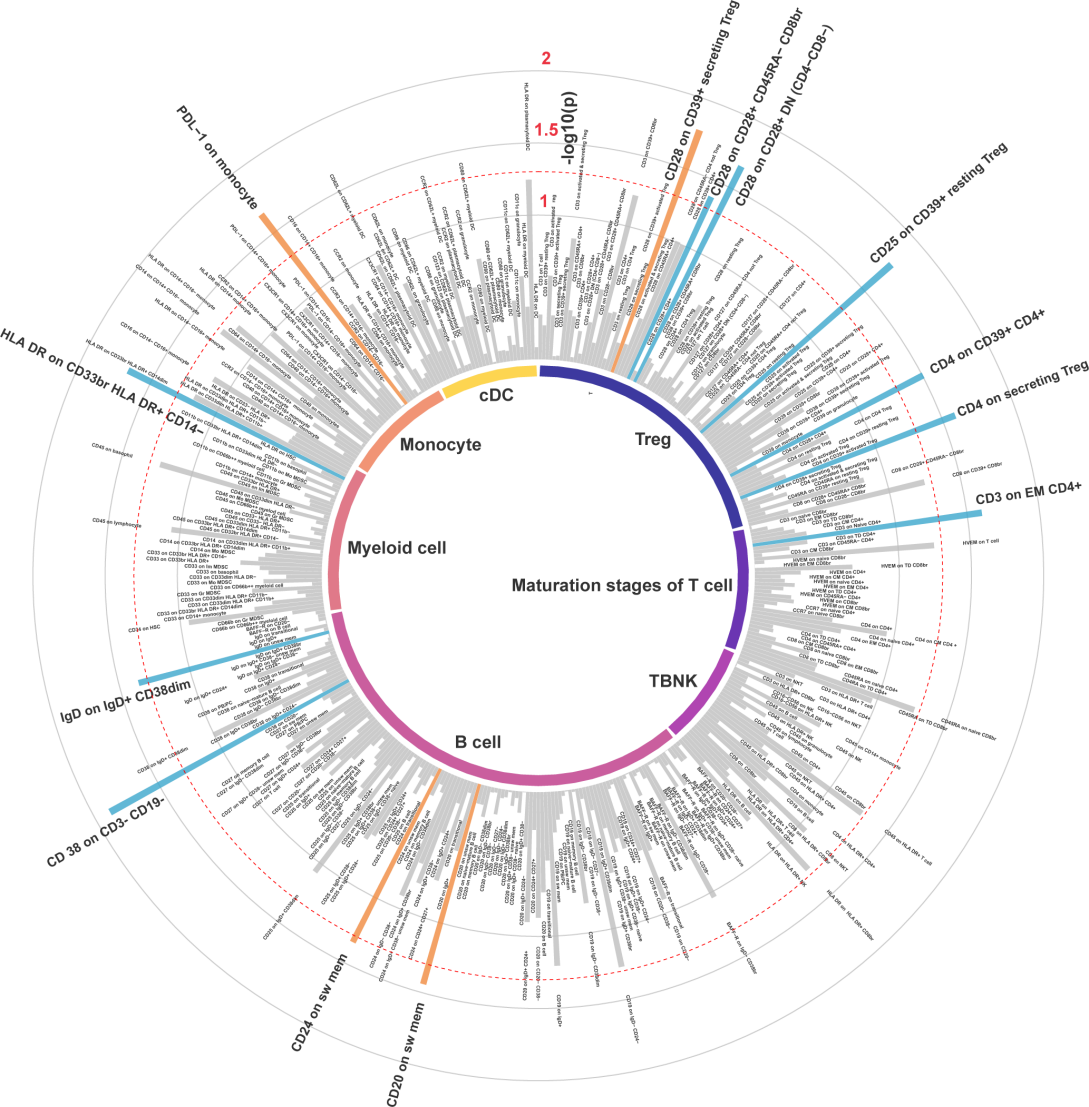
Inverse variance weighted estimates of the causal association between 389 immune cell surface antigens and post-stroke functional outcome. The red dashed line indicates the threshold of significance (*P<* 0.05). Orange bars represent deleterious mediators of post-stroke functional outcome, whereas blue bars represent protective mediators of post-stroke functional outcome.

In the IVW MR analysis of the expression levels of immune cell surface antigens and post-stroke functional outcome, 13 suggestive MFIs of surface antigens were identified, of which 4 were in the B cell panel, 1 in maturation stages of the T cell panel, 6 in the Treg panel, 1 in myeloid cell panel, and 1 in the monocyte panel (**Figure 2**).

**Figure 3:**
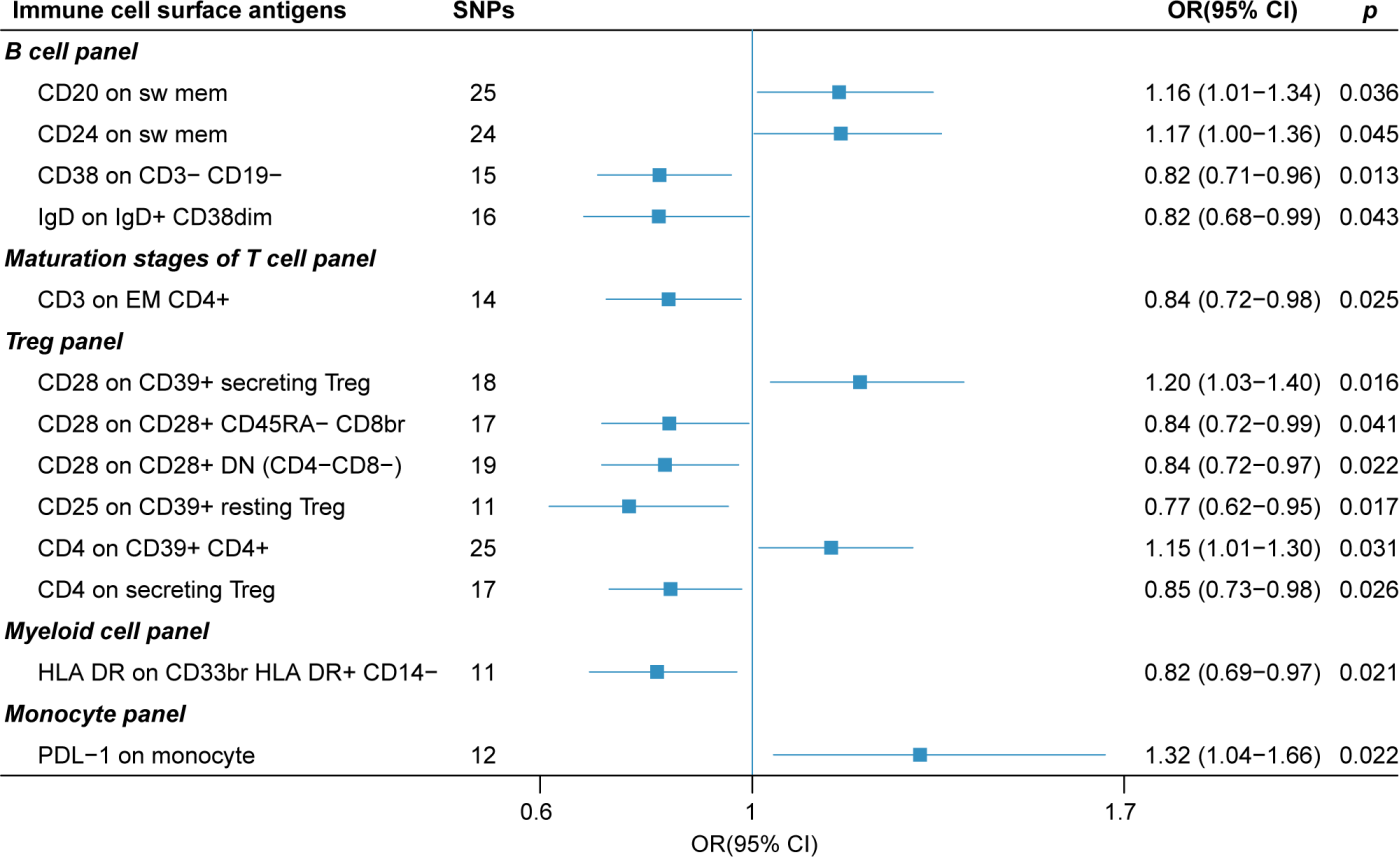
The forest plot of 13 immune cell surface antigens with functional outcome after ischemic stroke adjustment for baseline stroke severity (p for Inverse variance weighted method< 0.05).

The forest plot in **Figure 3** presents the IVW estimates of the associations between the levels of these immune cell surface antigens and post-stroke functional outcome.

Genetically elevated levels of surface antigens in the DC and TBNK panel were not strongly associated with post-stroke functional outcome (all *p >* 0.05). We also conducted a reverse MR analysis (IVW method), which did not reveal a causal effect of post-stroke functional outcome on these immune cell surface antigens, suggesting no reverse causal effect (**Supplementary Table S3**).

### 3.2 Sensitivity analyses

To ensure the robustness of our findings, we conducted multiple sensitivity analysis methods to assess the presence of potential pleiotropy in the results obtained from the MR analysis described above.

Our sensitivity analyses yielded consistent and reassuring results (**Table 2**). Specifically, we found no evidence of heterogeneity, as indicated by all p-values for Cochran’s Q test exceeding 0.05. This suggests a lack of substantial variability among the instrumental variables used in the MR analysis. Furthermore, our assessment of directional pleiotropy using the MR-PRESSO global test and MR-Egger intercept revealed no significant deviations from the IVW method. All p-values exceeded 0.05, indicating that the potential for pleiotropy did not substantially influence our findings. Additionally, the weighted median and MR-PRESSO methods produced effect estimates that were concordant with those obtained from the IVW method. This consistency reinforces the reliability of our results. However, it is worth noting that in the MR-Egger (slope) analysis, we observed that the effects of CD24 on switch memory B cell and CD3 on EM CD4+ maturation stages of T cell were estimated in the opposite direction compared to the results obtained from other MR analysis methods. This discrepancy suggests the need for a cautious interpretation of these particular associations.

**Table 2:** Sensitive analyses between 13 immune cell surface antigens and functional outcome after ischemic stroke (adjustment for baseline stroke severity).

MFIs of immune cell surface antigens	MR-Egger (slope)		weighted median		MR-PRESSO		MR-Egger (intercept)	MR-PRESSO global test	Cochran’s Q test
	OR (95% CI)	p	OR (95% CI)	p	OR (95% CI)	p	p	p	p
B cell panel									
**CD20 on sw mem**	1.11 (0.77-1.59)	0.59	1.28 (1.04-1.56)	0.02	1.16 (1.01-1.34)	0.046	0.77	0.49	0.47
**CD24 on sw mem**	0.96 (0.64-1.43)	0.84	1.08 (0.86-1.35)	0.53	1.17 (1.01-1.34)	0.042	0.31	0.64	0.65
**CD38 on CD3- CD19-**	0.90 (0.66-1.23)	0.52	0.79 (0.65-0.98)	0.03	0.82 (0.72-0.95)	0.015	0.55	0.68	0.66
**IgD on IgD+ CD38dim**	0.75 (0.44-1.28)	0.31	0.89 (0.70-1.13)	0.34	0.82 (0.68-0.99)	0.061	0.72	0.30	0.25
Maturation stages of T cell panel									
**CD3 on EM CD4+**	1.03 (0.76-1.40)	0.87	0.93 (0.76-1.13)	0.46	0.84 (0.73-0.97)	0.030	0.17	0.46	0.62
Treg panel									
**CD28 on CD39+ secreting Treg**	1.43 (1.05-1.96)	0.04	1.16 (0.94-1.43)	0.18	1.20 (1.06-1.37)	0.012	0.22	0.78	0.78
**CD28 on CD28+ CD45RA- CD8br**	0.88 (0.63-1.22)	0.44	0.81 (0.64-1.02)	0.08	0.84 (0.76-0.93)	0.004	0.80	0.99	0.99
**CD28 on CD28+ DN (CD4-CD8-)**	0.82 (0.60-1.12)	0.22	0.80 (0.63-1.00)	0.05	0.84 (0.75-0.94)	0.006	0.87	0.95	0.94
**CD25 on CD39+ resting Treg**	0.69 (0.44-1.09)	0.14	0.67 (0.49-0.91)	0.01	0.77 (0.62-0.95)	0.037	0.61	0.49	0.45
**CD4 on CD39+ CD4+**	1.26 (0.98-1.63)	0.09	1.14 (0.95-1.36)	0.15	1.15 (1.01-1.30)	0.042	0.42	0.35	0.32
**CD4 on secreting Treg**	0.82 (0.59-1.14)	0.25	0.89 (0.73-1.09)	0.26	0.85 (0.76-0.94)	0.009	0.84	0.92	0.91
Myeloid cell panel									
**HLA DR on CD33br HLA DR+ CD14-**	0.59 (0.33-1.04)	0.10	0.80 (0.64-1.01)	0.06	0.82 (0.71-0.94)	0.019	0.44	0.71	0.74
Monocyte panel									
**PDL-1 on monocyte**	1.60 (0.94-2.72)	0.12	1.32 (0.96-1.80)	0.09	1.32 (1.04-1.66)	0.043	0.26	0.35	0.31

**Figure 4:**
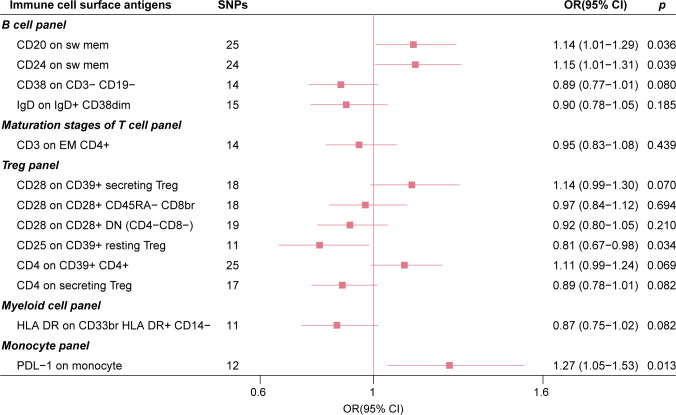
The forest plot of 13 immune cell surface antigens with functional outcome after ischemic stroke without adjustment for baseline stroke severity.

As a comparative analysis, we performed MR analysis based on GISCOME GWAS data without adjustment for baseline NIHSS. This analysis revealed that four immune cell surface antigens maintained suggestive causal associations with post-stroke functional outcome based on IVW estimates (**Figure 4**). Importantly, this comparison analysis showed no significant evidence of directional pleiotropy or global heterogeneity (**Supplementary Table S4**).

### 3.3 Two immune cell surface antigens were associated with the risk of stroke

To comprehensively evaluate our analyses, particularly in the context of prognostic implications, we investigated the potential presence of collider bias by examining the association of immune cell surface antigen levels with ischemic stroke risk using data from the MEGASTROKE dataset.

**Figure 5:**
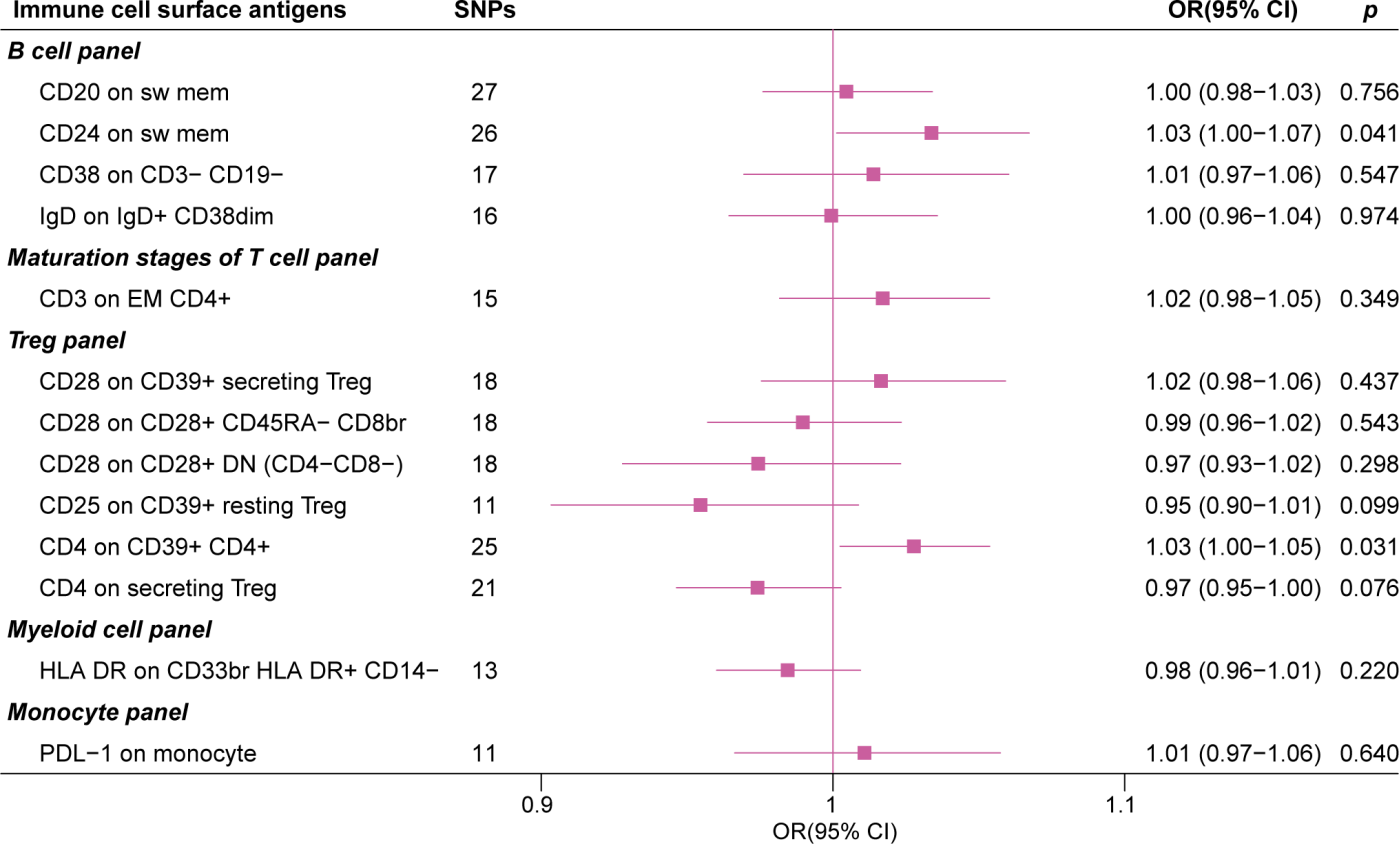
The forest plot of 13 immune cell surface antigens with risk of ischemic stroke.

The MR analysis outcome revealed that CD24 on switch memory B cell (OR = 1.03, 95% CI: 1.001-1.067, p = 0.041) and CD4 on CD39+ CD4+ Treg (OR = 1.03, 95% CI: 1.002-1.054, p = 0.031) were weakly associated with an increased risk of ischemic stroke (**Figure 5**). These associations raise the possibility that the observed relationships between these specific immune cell surface antigens and poor post-stroke functional outcome may, in part, be influenced by collider bias. However, it is essential to note that our assessment of collider bias suggests that any such bias, if present, is likely to be minimal. This indicates that while these immune cell surface antigens may have some impact on both ischemic stroke risk and post-stroke functional outcome, collider bias is unlikely to be a major driver of the observed associations.

## 4 Discussion

The clinical significance of post-stroke functional outcome lies in its impact on the overall quality of life and long-term prognosis of stroke survivors, which is critical in determining appropriate care and rehabilitation efforts (17). In addition, it can help predict the risk of future health complications such as falls, infections, and depression (2). Actively exploring immunological factors that affect stroke recovery and intervening can help patients maximize their recovery and regain independence after stroke. While numerous observational studies have provided substantial evidence of immune cell involvement in stroke onset and outcome, they suffer from inherent limitations, including uncontrollable biases and the heterogeneity of study metrics. Immune cell surface antigens can potentially unravel the precise roles of these immune cells following ischemic stroke. However, previous investigations have primarily overlooked the contribution of cell surface antigen biological functions in the context of stroke. This study marks the inaugural effort to explore the causal relationship between immune cell surface antigens and post-stroke functional outcome using MR analysis.

In the initial primary MR analysis, we identified 13 immune cell surface antigens associated with post-stroke functional outcome. Following rigorous sensitivity analyses and an assessment of collider bias, we ultimately identified three robust immune cell surface antigens with suggestive causal associations linked to post-stroke functional outcome. Specifically, elevated levels of CD20 on switched memory B cell and PDL-1 on monocyte were associated with poorer post-stroke functional outcome. In contrast, increased expression of CD25 on CD39+ resting Treg was linked to a more favorable post-stroke functional outcome (19).

CD20, encoded by MS4A1, is a non-glycosylated protein belonging to the membrane-spanning 4-domain family A (MS4A) protein family (25). Beginning with late pre-B lymphocytes, most B cells express CD20, and its expression is diminishing in terminally differentiated plasma cells. Therefore, CD20 can be used as a marker for developing B cells, and CD20-specific inhibitors are commonly used to treat B cell malignancies and autoimmune diseases (26). However, the precise biological function of CD20 and regulatory mechanisms remain elusive. Tedder TF et al. have suggested that CD20 might influence B cell proliferation and activation by modulating Ca2+ transmembrane transport (27). A case report demonstrated that CD20 deficiency reduced circulating memory B cell counts, impaired Ig isotype switching, and diminished IgG antibody levels (28). Upon activation, memory B cells undergo isotypic transformation from IgD/IgM to IgG/IgA/IgE (29). Thus, CD20 may be involved in the isotypic transformation of memory B cells. Our findings indicate that higher CD20 expression on switched memory B cells is associated with poor long-term outcome in patients with ischemic stroke. This suggests that CD20 expression levels may trigger the conversion of B cells from a specific state to pathogenic entities following ischemic stroke. Notably, the concept that B cells can contribute to central nervous system (CNS) pathology independently of antibody production has been discussed in the context of multiple sclerosis (30). B cells can release factors that disrupt the CNS, leading to oligodendrocyte and neuronal death. Targeting CD20 has proven effective in multiple sclerosis treatment (31).

Similarly, the expression level of PDL-1 on monocytes exhibited a similar effect. Accumulating evidence from animal models and patient studies suggests that ischemic stroke prompts the recruitment of circulating monocytes into the brain, where they differentiate into macrophages or dendritic cells, influencing ischemic injury progression (32, 33). Elevated monocyte counts have been associated with worse stroke outcome and greater stroke severity, making them potential predictive biomarkers for post-stroke functional outcome (34, 35). Our findings suggest that PDL-1 on monocytes may play a role in mediating unfavorable post-stroke functional outcome. PDL-1, the primary ligand for PD-1, is widely expressed in B cells, T cells, DCs, and monocytes, regulating immune function in these cell types (36). Bodhankar S. et al. have shown that homozygous knock-out (PDL-1-/-) mice had reduced monocyte infiltration, smaller infarct sizes in the ischemic hemisphere, and reduced activation status of splenic monocytes compared to wild-type (WT) mice, implying PDL-1’s involvement in exacerbating experimental stroke outcome (37). Combining these results with our analysis, it can be inferred that monocytes with high PDL-1 expression may be pivotal in controlling the adverse effects of ischemia.

On the other hand, our results suggest a beneficial role for increased CD25 expression on CD39+ resting Tregs in post-stroke functional outcome. CD25, the alpha-chain of the heterotrimer IL-2 receptor, also known as IL2Rα, is constitutively expressed at high levels in most Tregs. CD25 (α-chain), together with CD122 (β-chain) and CD132 (γ-chain), forms the functional IL-2 receptor (IL-2R), and of these three receptor chains, the binding affinity of CD25 for IL-2 is the highest (38). IL-2 signalling can affect Treg peripheral induction, lineage commitment, stability sustainability, and homeostasis, and CD25 expression is critical for IL-2 signalling to Treg (39, 40). Thus, the expression status of CD25 influences, to some extent, the immunomodulatory functions of Treg involved in re-establishing immune homeostasis and regulating inflammatory response after ischemic stroke. High CD25 expression influences the immunomodulatory functions of Tregs, contributing to immune homeostasis restoration and regulation of inflammatory reactions post-stroke. However, it is essential to note that these effects may be specific to certain Treg subpopulations. Elevated CD25 expression may support the survival and maintenance of resting Tregs, while CD39 surface expression is involved in the hydrolysis of extracellular ATP, essential for immunosuppressive function. These mechanisms likely play a crucial role in long-term immune homeostasis after stroke (41, 42).

Nonetheless, several limitations of this study should be acknowledged. Firstly, measuring immune cell surface antigen levels (MFIs) involves flow cytometry on peripheral blood samples, which can introduce time-dependent artifacts. These time-dependent effects were not considered in the current MR analysis. Additionally, MR estimates might introduce bias when comparing brain and blood, necessitating careful consideration of the tissue specificity of ischemic stroke. Secondly, the GISCOME database lacked outcome data for specific stroke subtypes, preventing an assessment of immune cell surface antigen relationships with functional outcome in different stroke subtypes. Moreover, a lack of available data for replication analysis may have reduced the persuasiveness of our results. Lastly, our MR analysis was limited to subjects of European ancestry, potentially limiting the generalizability of our findings to other populations.

## 5 Conclusion

This MR study offers compelling evidence that specific immune cell surface antigen levels are associated with adverse post-stroke functional outcome. CD20 on switched memory B cell, PDL-1 on monocyte, and CD25 on CD39+ resting Treg emerge as potential biomarkers and causal factors linked to post-stroke functional outcome. However, the underlying biological mechanisms require further exploration, and the potential of targeting these immune cell surface antigens as a therapeutic strategy to enhance post-stroke recovery warrants further investigation.

## Data availability statement

The raw data supporting the conclusions of this article will be made available by the authors, without undue reservation.

## Ethics statement

The author stated that no human studies are presented in the manuscript.

## Author contributions

WS: Conceptualization, Funding acquisition, Methodology, Project administration, Supervision, Writing – review & editing. JG: Formal analysis, Investigation, Methodology, Software, Visualization, Writing – original draft, Writing – review & editing. KW: Data curation, Formal analysis, Methodology, Software, Visualization, Writing – original draft, Writing – review & editing. YC: Formal analysis, Investigation, Methodology, Software, Writing – review & editing. XD: Conceptualization, Formal analysis, Supervision, Visualization, Writing – review & editing. GY: Conceptualization, Funding acquisition, Methodology, Project administration, Supervision, Writing – review & editing. CZ: Conceptualization, Funding acquisition, Project administration, Supervision, Writing – review & editing. LS: Conceptualization, Funding acquisition, Methodology, Project administration, Supervision, Visualization, Writing – review & editing. ZF: Methodology, Project administration, Writing – review & editing.

## References

[1] Roth GA Mensah GA Johnson CO Addolorato G Ammirati E Baddour LM. Global burden of cardiovascular diseases and risk factors, 1990-2019: update from the GBD 2019 study. J Am Coll Cardiol. (2020) 76:2982–3021. doi: 10.1016/j.jacc.2020.11.010 33309175 PMC7755038

[2] Westendorp WF Dames C Nederkoorn PJ Meisel A. Immunodepression, infections, and functional outcome in ischemic stroke. Stroke. (2022) 53:1438–48. doi: 10.1161/STROKEAHA.122.038867 35341322

[3] Lv M Zhang Z Cui Y. Unconventional T cells in brain homeostasis, injury and neurodegeneration. Front Immunol. (2023) 14:1273459. doi: 10.3389/fimmu.2023.1273459 37854609 PMC10579804

[4] Wang H Zhang S Xie L Zhong Z Yan F. Neuroinflammation and peripheral immunity: Focus on ischemic stroke. Int Immunopharmacol. (2023) 120:110332. doi: 10.1016/j.intimp.2023.110332 37253316

[5] Li Y Wang Y Yao Y Griffiths BB Feng L Tao T. Systematic study of the immune components after ischemic stroke using cyTOF techniques. J Immunol Res. (2020) 2020:9132410. doi: 10.1155/2020/9132410 32908941 PMC7474762

[6] Krishnan S O'Boyle C Smith CJ Hulme S Allan SM Grainger JR. A hyperacute immune map of ischaemic stroke patients reveals alterations to circulating innate and adaptive cells. Clin Exp Immunol. (2021) 203:458–71. doi: 10.1111/cei.13551 PMC7874838 33205448

[7] Wang M Thomson AW Yu F Hazra R Junagade A Hu X. Regulatory T lymphocytes as a therapy for ischemic stroke. Semin Immunopathol. (2023) 45:329–46. doi: 10.1007/s00281-022-00975-z PMC10239790 36469056

[8] Wang HY Ye JR Cui LY Chu SF Chen NH. Regulatory T cells in ischemic stroke. Acta Pharmacol Sin. (2022) 43:1–9. doi: 10.1038/s41401-021-00641-4 33772140 PMC8724273

[9] Li S Huang Y Liu Y Rocha M Li X Wei P. Change and predictive ability of circulating immunoregulatory lymphocytes in long-term outcomes of acute ischemic stroke. J Cereb Blood Flow Metab. (2021) 41:2280–94. doi: 10.1177/0271678X21995694 PMC8393304 33641517

[10] Zhang D Ren J Luo Y He Q Zhao R Chang J. T cell response in ischemic stroke: from mechanisms to translational insights. Front Immunol. (2021) 12:707972. doi: 10.3389/fimmu.2021.707972 34335623 PMC8320432

[11] Smith GD Ebrahim S. ‘Mendelian randomization’: can genetic epidemiology contribute to understanding environmental determinants of disease? Int J Epidemiol. (2003) 32:1–22. doi: 10.1093/ije/dyg070 12689998

[12] Davey Smith G Holmes MV Davies NM Ebrahim S. Mendel’s laws, Mendelian randomization and causal inference in observational data: substantive and nomenclatural issues. Eur J Epidemiol. (2020) 35:99–111. doi: 10.1007/s10654-020-00622-7 32207040 PMC7125255

[13] Hu S Lin Z Hu MJ Tan JS Guo TT Huang X. Causal relationships of circulating amino acids with cardiovascular disease: a trans-ancestry Mendelian randomization analysis. J Transl Med. (2023) 21:699. doi: 10.1186/s12967-023-04580-y 37805555 PMC10559604

[14] Orrù V Steri M Sidore C Marongiu M Serra V Olla S. Complex genetic signatures in immune cells underlie autoimmunity and inform therapy. Nat Genet. (2020) 52:1036–45. doi: 10.1038/s41588-020-0684-4 PMC8517961 32929287

[15] Maguire JM Bevan S Stanne TM Lorenzen E Fernandez-Cadenas I Hankey GJ. GISCOME - Genetics of Ischaemic Stroke Functional Outcome network: A protocol for an international multicentre genetic association study. Eur Stroke J. (2017) 2:229–37. doi: 10.1177/2396987317704547 PMC6454830 31008316

[16] Söderholm M Pedersen A Lorentzen E Stanne TM Bevan S Olsson M. Genome-wide association meta-analysis of functional outcome after ischemic stroke. Neurology. (2019) 92:e1271–83. doi: 10.1212/WNL.0000000000007138 PMC6511098 30796134

[17] Lindgren A Maguire J. Stroke recovery genetics. Stroke. (2016) 47:2427–34. doi: 10.1161/STROKEAHA.116.010648 27515845

[18] Tao C Yuan Y Xu Y Zhang S Wang Z Wang S. Role of cognitive reserve in ischemic stroke prognosis: A systematic review. Front Neurol. (2023) 14:1100469. doi: 10.3389/fneur.2023.1100469 36908598 PMC9992812

[19] Ankolekar S Renton C Sare G Ellender S Sprigg N Wardlaw JM. Relationship between poststroke cognition, baseline factors, and functional outcome: data from “efficacy of nitric oxide in stroke” trial. J Stroke Cerebrovasc Dis. (2014) 23:1821–9. doi: 10.1016/j.jstrokecerebrovasdis.2014.04.022 24957311

[20] Malik R Chauhan G Traylor M Sargurupremraj M Okada Y Mishra A. Multiancestry genome-wide association study of 520,000 subjects identifies 32 loci associated with stroke and stroke subtypes. Nat Genet. (2018) 50:524–37. doi: 10.1038/s41588-018-0058-3 PMC5968830 29531354

[21] Bowden J Davey Smith G Haycock PC Burgess S. Consistent estimation in Mendelian randomization with some invalid instruments using a weighted median estimator. Genet Epidemiol. (2016) 40:304–14. doi: 10.1002/gepi.21965 PMC4849733 27061298

[22] Bowden J Davey Smith G Burgess S. Mendelian randomization with invalid instruments: effect estimation and bias detection through Egger regression. Int J Epidemiol. (2015) 44:512–25. doi: 10.1093/ije/dyv080 PMC4469799 26050253

[23] Verbanck M Chen CY Neale B Do R. Detection of widespread horizontal pleiotropy in causal relationships inferred from Mendelian randomization between complex traits and diseases. Nat Genet. (2018) 50:693–8. doi: 10.1038/s41588-018-0099-7 PMC6083837 29686387

[24] Hemani G Zheng J Elsworth B Wade KH Haberland V Baird D. The MR-Base platform supports systematic causal inference across the human phenome. Elife. (2018) 7:e34408. doi: 10.7554/eLife.34408 29846171 PMC5976434

[25] Tedder TF Klejman G Schlossman SF Saito H. Structure of the gene encoding the human B lymphocyte differentiation antigen CD20 (B1). J Immunol. (1989) 142:2560–8. doi: 10.4049/jimmunol.142.7.2560 2466899

[26] Pavlasova G Mraz M. The regulation and function of CD20: an “enigma” of B-cell biology and targeted therapy. Haematologica. (2020) 105:1494–506. doi: 10.3324/haematol.2019.243543 PMC7271567 32482755

[27] Tedder TF Schlossman SF. Phosphorylation of the B1 (CD20) molecule by normal and Malignant human B lymphocytes. J Biol Chem. (1988) 263:10009–15. doi: 10.1016/S0021-9258(19)81618-6 2454914

[28] Kuijpers TW Bende RJ Baars PA Grummels A Derks IA Dolman KM. CD20 deficiency in humans results in impaired T cell-independent antibody responses. J Clin Invest. (2010) 120:214–22. doi: 10.1172/JCI40231 PMC2798692 20038800

[29] Maity PC Blount A Jumaa H Ronneberger O Lillemeier BF Reth M. B cell antigen receptors of the IgM and IgD classes are clustered in different protein islands that are altered during B cell activation. Sci Signal. (2015) 8:ra93. doi: 10.1126/scisignal.2005887 26373673

[30] Häusser-Kinzel S Weber MS. The role of B cells and antibodies in multiple sclerosis, neuromyelitis optica, and related disorders. Front Immunol. (2019) 10:201. doi: 10.3389/fimmu.2019.00201 30800132 PMC6375838

[31] Jain RW Yong VW. B cells in central nervous system disease: diversity, locations and pathophysiology. Nat Rev Immunol. (2022) 22:513–24. doi: 10.1038/s41577-021-00652-6 PMC8667979 34903877

[32] Chiba T Umegaki K. Pivotal roles of monocytes/macrophages in stroke. Mediators Inflammation. (2013) 2013:759103. doi: 10.1155/2013/759103 PMC3568889 23431245

[33] Miró-Mur F Pérez-de-Puig I Ferrer-Ferrer M Urra X Justicia C Chamorro A. Immature monocytes recruited to the ischemic mouse brain differentiate into macrophages with features of alternative activation. Brain Behav Immun. (2016) 53:18–33. doi: 10.1016/j.bbi.2015.08.010 26275369

[34] Liberale L Montecucco F Bonaventura A Casetta I Seraceni S Trentini A. Monocyte count at onset predicts poststroke outcomes during a 90-day follow-up. Eur J Clin Invest. (2017) 47:702–10. doi: 10.1111/eci.12795 28783210

[35] Nadareishvili Z Luby M Leigh R Shah J Lynch JK Hsia AW. An MRI hyperintense acute reperfusion marker is related to elevated peripheral monocyte count in acute ischemic stroke. J Neuroimaging. (2018) 28:57–60. doi: 10.1111/jon.12462 28722240 PMC5760433

[36] Sun Y Tan J Miao Y Zhang Q. The role of PD-L1 in the immune dysfunction that mediates hypoxia-induced multiple organ injury. Cell Commun Signal. (2021) 19:76. doi: 10.1186/s12964-021-00742-x 34256773 PMC8276205

[37] Bodhankar S Chen Y Vandenbark AA Murphy SJ Offner H. PD-L1 enhances CNS inflammation and infarct volume following experimental stroke in mice in opposition to PD-1. J Neuroinflamm. (2013) 10:111. doi: 10.1186/1742-2094-10-111 PMC3846120 24015822

[38] Peng Y Tao Y Zhang Y Wang J Yang J Wang Y. CD25: A potential tumor therapeutic target. Int J Cancer. (2023) 152:1290–303. doi: 10.1002/ijc.34281 36082452

[39] Lykhopiy V Malviya V Humblet-Baron S Schlenner SM. “IL-2 immunotherapy for targeting regulatory T cells in autoimmunity”. Genes Immun. (2023) 24:248–62. doi: 10.1038/s41435-023-00221-y PMC10575774 37741949

[40] Boyman O Sprent J. The role of interleukin-2 during homeostasis and activation of the immune system. Nat Rev Immunol. (2012) 12:180–90. doi: 10.1038/nri3156 22343569

[41] Li Y Li X Geng X Zhao H. The IL-2A receptor pathway and its role in lymphocyte differentiation and function. Cytokine Growth Factor Rev. (2022) 67:66–79. doi: 10.1016/j.cytogfr.2022.06.004 35803834

[42] Timperi E Barnaba V. CD39 regulation and functions in T cells. Int J Mol Sci. (2021) 22(15):8068. doi: 10.3390/ijms22158068 34360833 PMC8348030

